# Neuropsychiatric symptoms and clinical characteristics of survivors with colloid cysts

**DOI:** 10.1186/s13023-025-03905-y

**Published:** 2025-08-11

**Authors:** Amanda Onoichenco, Tanveen Dhallu, Sarah Kabariti, Qiushuang Li, David Harter, Cinthi Pillai, Claire Snyman, Recai Yucel, Deborah Gustafson

**Affiliations:** 1 College of Medicine, State University of New York Downstate Health Sciences University, Brooklyn, NY USA; 2 Department of Neurology, State University of New York Downstate Health Sciences University, 450 Clarkson Avenue, Brooklyn, NY 11203 USA; 3 Department of Emergency Medicine, Maimonides Medical Center, Brooklyn, NY USA; 4 Department of Emergency Medicine, State University of New York Downstate Health Sciences University, Brooklyn, NY USA; 5 Department of Epidemiology and Biostatistics, Temple University, Philadelphia, PA USA; 6 Department of Neurology, New York University, New York, USA; 7 Two Steps Forward, Vancouver, BC Canada

**Keywords:** Colloid cyst, Foramen of Monro, Third ventricle, Brain tumor, Neurology, Neuropsychiatry, Psychiatry, Hydrocephalus

## Abstract

**Background:**

Colloid cysts are rare, benign brain tumors often located in the third ventricle or near the foramen of Monro. They can evoke neuropsychiatric and physical symptoms including migraine, visual changes, memory loss, and sudden loss of consciousness. They are associated with high mortality due to late moderate-to-severe symptom presentation and limited access to neurological and/or neurosurgical expertise. The Colloid Cyst Symptoms Survey (CCSS) was designed and administered anonymously using REDCap and posted to the Colloid Cyst Survivors Facebook group for 6 months in 2022. The CCSS queried about sociodemographic factors, personal history of a colloid cyst, age of cyst diagnosis, neuropsychiatric and physical symptoms/signs before and after surgery, procedure type if their cyst was removed, and follow-up with neurological, neuropsychiatric or psychological services. Psychiatric symptoms within the last two weeks were assessed using the DSM-5 Self-Rated Level 1 Cross-Cutting Symptom Measure—Adult assessing 13 psychiatric domains (American Psychiatric Association).

**Results:**

Participants included 225 adults who were U.S citizens with a personal history of a colloid cyst. The majority were female (71.3%) and White (94.0%). Age of diagnosis occurred between 12 and 75 years old, median age 42 years. On average, patients reported 2 pre-clinical symptoms leading to diagnosis, most commonly migraine (48.9%) and aura (30.7%). Hydrocephalus was reported by 56.9%. In patients who underwent surgical removal of their colloid cyst, craniotomy (53.9%) was more common than endoscopic removal (42.6%). Common conditions and physical complications persisting after surgery included changes in energy level (*N* = 68), memory problems (*N* = 71), and anxiety (*N* = 46); higher prevalence of post-surgical complications were observed in the hydrocephalus and craniotomy groups. The DSM-5 screener identified areas of concern including memory, anxiety, somatic symptoms, sleep difficulties, anger, depressive symptoms, suicidality, and substance use. Despite this only ~ 1/10 patients received follow-up with psychiatrists or psychologists.

**Conclusions:**

To our knowledge this is the largest cross-sectional study querying clinical characteristics among colloid cyst survivors. Persisting neuropsychiatric symptoms were reported to be high. People experiencing brain surgery, even for benign tumors, need to be assessed for neuropsychiatric morbidity.

## Background

Colloid cysts (CC) are benign, slow-growing, brain tumors located in the center of the brain [1].They are assumed to be fetal in origin and have a smooth rind (outer sac) filled with a gelatinous material called colloid. This colloid can range from being very fluid to having a nearly solid core. Colloid cysts belong to the category of rare diseases per Orphanet and occur in only ~ 3 people per million [2, 3]. They are typically diagnosed in adults in their fourth, fifth or sixth decades of life [1, 4]. They tend to occur in the third ventricle near the foramen of Monro, at the junction of the paired lateral ventricles. As a result of this location, they can cause blockage of cerebrospinal fluid (CSF) flow, which leads to hydrocephalus (excess brain CSF, also called ‘water on the brain’). Hydrocephalus can lead to many of the symptoms typically reported by patients with colloid cysts – adult-onset migraine, visual changes, fatigue, memory difficulties and occasionally, loss of consciousness, including very brief ‘sudden drops’, or coma [4].

While colloid cysts are relatively uncommon, in symptomatic patients they are associated with mortality rates as high as 12% [5]. Given the rarity of colloid cysts, few large studies assessing mortality rate exist and those that do are somewhat dated. High mortality occurs because of the incipient nature of cyst development, late moderate-to-severe symptom presentation in many people, and the lack of available medical expertise and brain imaging to make the diagnosis. In addition, medical coding systems, such as ICD-10 (G93.0) or CPT (62162) codes, are inadequate for determining the prevalence of this type of brain tumor.

Due to the rarity of this cyst, there is little known about preclinical presentation, risk factors, correlates and/or sequelae. The literature is sparse, but provides some background, primarily case reports, on neuropsychiatric [6, 7] and physical symptoms [1, 4, 5, 8, 9]. More and better data are needed to support improved management of those experiencing these brain tumors that are often removed under acute, life-threatening circumstances [10]. Therefore, we created a cross-sectional survey to assess sociodemographic factors, colloid cyst characteristics, and neuropsychiatric and physical symptoms among adults with a colloid cyst diagnosis, both pre-surgery and post-cyst removal.

## Methods

### Aim

The aim of this study was to characterize physical and neuropsychiatric symptoms and signs, as well as diagnosis and treatment of people diagnosed with a colloid cyst.

### Design

The study design was an anonymous online cross-sectional Colloid Cyst Survivor Survey (CCSS) among members of the Colloid Cyst Survivors Facebook group.

### Setting

The CCSS was accessed via a link offered online to members of the Facebook group for six months (May-November 2022). It was designed and administered anonymously using REDCap. Participants could complete any items of the survey they desired. The CCSS was conducted by a New York City research team at the State University of New York (SUNY) Downstate Health Sciences University (DHSU) in Brooklyn, Maimonides Medical Center in Brooklyn, and New York University in Manhattan, New York City.

### Characteristics of participants

Only people *≥* 18 years, who were citizens and resident in the U.S., and with history of or current colloid cyst could participate.

### Description of materials

The CCSS queried about sociodemographic factors (year of birth, sex assigned at birth, race, ethnicity), personal history of colloid cyst including age of cyst diagnosis, neuropsychiatric and physical symptoms and signs experienced before and after surgery to remove their cyst, procedure type, and healthcare follow-up. Psychiatric symptoms within the last two weeks were assessed using the DSM-5 Self-Rated Level 1 Cross-Cutting Symptom Measure—Adult assessing 13 psychiatric domains from the American Psychiatric Association [11]. This tool contains 23 questions across the 13 psychiatric domains including depression, anger, mania, anxiety, somatic symptoms, suicidal ideation, psychosis, sleep problems, memory, repetitive thoughts and behaviors, dissociation, personality functioning, and substance use. There are 1–3 questions for each domain. The questions ask the patient to reflect on how often they have experienced a symptom and answer according to the following scale: None (0) – Not at all; Slight [1] – Rare, less than a day or two; Mild [2] – Several days; Moderate [3] – More than half the days; Severe [4] – Nearly every day. We focused on individual symptoms rather than domains flagged for follow-up, with scores of mild [2] and above on an individual question indicating cause for concern; the threshold is lower with a score of slight [1] for domains including substance use, suicidal ideation, and psychosis.

The CCSS can be accessed here: https://redcap.downstate.edu/redcap/surveys/?s=C7FHHF7RXX9MEKME.

### Statistical analyses and methods

Descriptive statistics were calculated to describe the study sample. Medians and standard deviations were calculated for continuous variables. Frequencies and percents were calculated for categorical variables. One-way ANOVA was used to compare the average number of symptoms by sex, hydrocephalus status, and cyst removal procedure type. Results were deemed significant using a two-sided p-value < 0.05. Statistical analyses were performed using r (R version 4.2.2 (2022-10-31 ucrt) -- “Innocent and Trusting” Copyright (C) 2022 The R Foundation for Statistical Computing).

## Results

Prior to being able to access the CCSS, respondents had to respond that they had U.S citizenship and resided in the U.S., were an adult (*≥* 18 years old), and had personal history of a colloid cyst. The number eligible totaled *N* = 225. Given that respondents could skip any items on the survey, the denominator for each CCSS variable varies (e.g., see Table 1). Percentages were reported based on those who responded individually to each item (not the total N). Most respondents indicated female sex at birth (71.3%, 134/188) and White race (94.0%, 173/184). Colloid cyst diagnosis occurred between age 12–75 years (y) (median 42y) with an average of two pre-clinical symptoms, but this ranged widely from zero to 17 pre-clinical symptoms. Approximately half of respondents reported migraine (48.9%), followed by aura (30.7%), “other” (26.2%), vertigo (25.3%), memory problems (24.9%), and/or fatigue (24.0%) as reasons for cyst discovery. Other reasons are provided in Table 2. Hydrocephalus accompanied the cyst in over 50% of cases (56.9%). Of 225 participants, 115 reported type of cyst removal (51.1%) with the majority undergoing craniotomy (53.9%) compared to endoscopic removal (42.6%) (Table 1). While the majority of CCSS participants reported regular follow-up with a neurosurgeon or neurologist (83.5%, 121/145), ∼only 1 in 10 patients was followed by a psychiatrist or psychologist (Table 1).

**Table 1:** Characteristics of adult colloid cyst survivors

	Characteristic	Median (range) or *N* (%)	*N* Responses
1	Year of birth	1971(1945, 2007)	155
2	Female sex assigned at birth	134(71.3)	188
3	Ethnicity		62
	Hispanic or Latino	4(6.5)	
	NOT Hispanic or Latino	55(88.7)	
	Unknown/Not reported	3(4.8)	
4	Race		184
	American Indian/ Alaska Native	4(2.2)	
	Asian	3(1.6)	
	Black or African American	1(0.5)	
	White	173(94.0)	
	Other	3(1.6)	
5	Age at first colloid cyst diagnosis, years	42(12–75)	174
6	Number of pre-clinical symptoms	2(0–17)	
7	Brain imaging technique(s) used to diagnose of monitor the cyst*		
	CAT scan/CT	98	
	MRI 1.5T	20	
	MRI 3T	10	
	MRI (magnet strength unknown)	120	
	I don’t know	6	
8	Hydrocephalus present	87(56.9)	153
9	Cyst size (cm)	1.4(0.07–19)	120
10	Type of cyst removal procedure (if removed)		115
	Minimally invasive endoscopy	49(42.6)	
	Craniotomy	62(53.9)	
	Other	4(3.5)	
11	Regular follow-up with*		
	Neurosurgeon or neurologist?	121	
	Psychiatrist or neuropsychiatrist?	14	
	Neuropsychologist or psychologist?	16	

*Since this portion of the CCSS was a checklist, the denominator is unknown

**Table 2:** Pre-clinical symptoms experienced leading up to a colloid cyst diagnosis

	Pre-clinical Symptoms	N*
1	Anxiety	20
2	Chest pain, rapid heart rate	10
3	Migraine	110
4	Aura (flashes of light, blind spots, other vision changes)	69
5	Vertigo (‘spinning’, loss of balance)	57
6	Memory problem	56
7	Cognitive impairments other than memory problems	28
8	Amnesia	4
9	Fatigue or needing to lie down	54
10	Physical performance difficulties, e.g., changes in exercise routine	16
11	Apathy (lack of interest or concern)	18
12	Depression	20
13	Social withdrawal	10
14	Delusions	1
15	Mania (periods of excitement or overactivity)	2
16	Felt like early aging	14
17	Incontinence	12
18	Insomnia	17
19	Restlessness	7
20	Sexual disinhibition	8
21	Personality changes	21
22	Seizures	5
23	Nausea or upset stomach	43
24	Vomiting	25
25	‘Sudden drop’ or falling down without warning	25
26	Other	59

*Since this portion of the CCSS was a checklist, the denominator is unknown

Since removal, the most frequently reported conditions were memory problems (*N* = 71) and anxiety (*N* = 46). Changes in energy level (*N* = 68), sleep (*N* = 58), physical activity (*N* = 39), body weight (*N* = 34), sex life (*N* = 27), gait/walking (*N* = 20), or other (*N* = 4) were reported. The primary physical complication of cyst removal was headache/migraine (*N* = 37). See Table 3. 10% reported repeated surgery for their cyst. Among a subset of participants responding to procedure type, there was a higher average number of pre-clinical symptoms among those who underwent endoscopic removal (*p* = 0.05). Additionally, there was a higher number of post-surgical conditions and physical complications in the hydrocephalus group (*p* = 0.000 and *p* = 0.001, respectively). Those who experienced craniotomy had more post-surgical sequelae (*p* = 0.006). No sex differences in the number of pre- and post-surgical symptoms were identified.

**Table 3:** Symptoms, conditions, and changes experienced by colloid cyst survivors after cyst removal surgery

	Post-Surgical Sequelae	N*
	Diagnosed with conditions such as	
1	Depression	36
2	Anxiety	46
3	Mania	4
4	Inhibition	16
5	Memory problems	71
6	Confusion ‘jumbled brain’	41
7	Dementia	4
8	Attention Deficit Disorder	9
9	Restlessness	13
10	General Mood Disorder	12
11	Post-Traumatic Stress Disorder (PTSD)	24
12	Other	14
	Experienced physical complications including	
1	Eye problems (e.g., retinal hemorrhage, optic nerve inflammation, double vision, vision loss/gain, floaters)	22
2	Infections due to surgery	1
3	Shunt inserted to drain fluid	11
4	Hematoma or collection of blood in the brain	3
5	Stroke or TIA (mini strokes)	4
6	Changes in hearing (e.g. loss or gain, tinnitus)	20
7	Headache or migraine	37
8	Other	18
	Changes in	
1	Physical activity or exercise habits	39
2	Sleep	58
3	Sex life	27
4	Body weight	34
5	Energy level	68
6	Gait or walking	20
7	Other	4

*Since this portion of the CCSS was a checklist, the denominator is unknown

Approximately 130 participants completed the DSM-5 screener (Table 4). Within the previous two weeks of completing the CCSS, the most experienced symptoms were in the domains of memory (*N* = 67), anxiety (*N* = 67) “feeling nervous, anxious, frightened, worried, or on edge” and avoiding situations that made them anxious (*N* = 63), and somatic symptoms (*N* = 65). There were also many reports of difficulty sleeping (*N* = 61), depression (*N* = 59 “feeling down, depressed, or hopeless”), and anger (*N* = 59). Of note, 21 individuals indicated suicidal ideation with thoughts of self-harm and 19 individuals drank at least 4 alcoholic drinks in a day. Those who experienced craniotomy had a greater number of concerning symptoms on the DSM-5 screener (*p* = 0.005).

A variety of self-medication strategies were reported, and the most common were nutritional or dietary supplements (*N* = 39), followed by meditation (*N* = 24), massage (*N* = 21), over the counter medications such as Sudafed or allergy medications containing pseudoephedrine (*N* = 20), smoking cigarettes or cigars/vaping (*N* = 15), alcohol (*N* = 12), cannabis or ‘weed’ (*N* = 7), and other (*N* = 13).

**Table 4:** Colloid cyst survivor responses to the DSM-5 screener by symptom

	DSM-5 Screener	*N* (%) meeting criteria for follow-up	*N* Responses
I. Depression	1. Little interest or pleasure in doing things?	52(40.6)	128
	2. Feeling down, depressed, or hopeless?	59(45.4)	130
II. Anger	3. Feeling more irritated, grouchy, or angry than usual?	59(45.7)	129
III. Mania	4. Sleeping less than usual, but still have a lot of energy?	39(30.5)	128
	5. Starting lots more projects than usual or doing more risky things than usual?	21(16.2)	130
IV. Anxiety	6. Feeling nervous, anxious, frightened, worried, or on edge?	67(51.5)	130
	7. Feeling panic or being frightened?	40(31.0)	129
	8. Avoiding situations that make you anxious?	63(48.8)	129
V. Somatic symptoms	9. Unexplained aches and pains (e.g., head, back, joints, abdomen, legs)?	65(50.4)	129
	10. Feeling that your illnesses are not being taken seriously enough?	51(39.8)	128
VI. Suicidal ideation	11. Thoughts of actually hurting yourself?	21(16.3)	129
VII. Psychosis	12. Hearing things other people couldn’t hear, such as voices even when no one was around?	13(10.2)	128
	13. Feeling that someone could hear your thoughts, or that you could hear what another person was thinking?	4(3.1)	129
VIII. Sleep problems	14. Problems with sleep that affected your sleep quality over all?	61(47.3)	129
IX. Memory	15. Problems with memory (e.g., learning new information) or with location (e.g., finding your way home)?	67(51.5)	130
X. Repetitive thoughts and behaviors	16. Unpleasant thoughts, urges, or images that repeatedly enter your mind?	24(18.5)	130
	17. Feeling driven to perform certain behaviors or mental acts over and over again?	16(12.4)	129
XI. Dissociation	18. Feeling detached or distant from yourself, your body, your physical surroundings, or your memories?	35(26.9)	130
XII. Personality Functions	19. Not knowing who you really are or what you want out of life?	33(25.6)	129
	20. Not feeling close to other people or enjoying your relationships with them?	36(27.9)	129
XIII. Substance use	21. Drinking at least 4 drinks of any kind of alcohol in a single day?	19(14.7)	129
	22. Smoking any cigarettes, a cigar, or pipe, or using snuff or chewing tobacco?	20(15.6)	128
	23. Using any of the following medicines ON YOUR OWN, that is, without a doctor’s prescription, in greater amounts or longer than prescribed [e.g., painkillers (like Vicodin), stimulants (like Ritalin or Adderall), sedatives or tranquilizers (like sleeping pills or Valium), or drugs like marijuana, cocaine or crack, club drugs (like ecstasy), hallucinogens (like LSD), heroin, inhalants or solvents (like glue), or methamphetamine (like speed)]?	9(7.0)	129

## Discussion

While colloid cysts are rare, responsible for less than 2% of primary brain tumors, the associated complications including hydrocephalus, brain herniation, and even death are a looming threat to the patient. Given the low reported prevalence of colloid cysts, the neuropsychiatric impact of such a diagnosis and subsequent surgery or treatment are not well understood [4]. Just as the etiology remains unknown, the population impacted is not well-described. A stronger characterization may bolster diagnosis, especially in patients with neurological and psychiatric symptoms or diagnoses. Due to their rarity, the paucity of reported cases limits proper prognostication and management including indications for surgery and risk for developing hydrocephalus [12].

Existing literature suggests that in adults, symptomatic presentation of colloid cysts usually occurs after the 3rd decade of life, consistent with our finding of an average age of 42 years old at diagnosis [13]. In contrast to existing literature which suggests a male sex predominance, most of our participants were White women which adds to the knowledge base [14, 15]. One potential explanation is higher engagement of women in this social media group as women are more inclined to turn to the internet to find answers to their health related questions [16].

Despite their non-malignant nature, colloid cysts may present with significant neuropsychiatric symptoms and signs that disrupt daily functioning most commonly including headache, vision disturbance, memory deficits, and psychiatric presentations. As self-reported by patients in this study, the most common symptoms leading to diagnosis were migraine in almost half of patients followed by aura, vertigo and memory problems. Headaches associated with a colloid cyst may be intermittent and positional in nature (worsening when upright) [10]. For physicians, in combination with patient profile (age, history of these symptoms) perhaps these should be herald signs prompting neuroimaging on initial evaluation or throughout the clinical assessment to lead to earlier diagnosis. The symptoms commonly associated with colloid cysts can often be explained by obstruction of CSF. The cyst may obstruct flow through the interventricular foramen resulting in dilation of the ventricles and increased intracranial pressure on surrounding brain areas [10]. On average there is a higher burden of morbidity and mortality by colloid cysts when accompanied by hydrocephalus (when the foramen of Monro is occluded). It is postulated that acute obstruction may explain the rare occurrence of sudden deterioration, however it is difficult to accurately assess the mechanism through which colloid cysts are linked to cases of sudden death (*Beaumont 2016*). Our findings showed hydrocephalus occurred in ~ 56% of colloid cysts patients, consistent with existing literature which estimates that approximately half of symptomatic patients present with obstructive hydrocephalus [12]. However, other case studies report colloid cyst-related symptoms in the absence of hydrocephalus and resolution following removal, perhaps due to diencephalon compression [17]. Review of the literature suggests anywhere from 29 − 58% of colloid cysts are asymptomatic and diagnosed incidentally when neuroimaging is obtained, most commonly for head trauma, headache, and neurological conditions like “stroke, peripheral neuropathy, and amyotrophic lateral sclerosis” [12, 13]. Even when found incidentally in asymptomatic patients and managed conservatively, a colloid cyst should necessitate close follow-up as 5–15% may progress and require surgical intervention within 5 years of diagnosis [18].

Colloid cysts may be managed with conservative, surgery-free approaches such as serial imaging. Alternatively, surgical options including more invasive transcortical and transcallosal microsurgical approaches or minimally invasive procedures such as stereotactic aspiration and endoscopic colloid cyst excision may be used. When microsurgical craniotomy and endoscopic approaches are compared, the microsurgical approach is more successful in achieving complete cyst removal with higher rates of total resection (96.0-96.8%) and lower recurrence rates (0.98–1.48%) than endoscopic groups (58.2–78.5% total resection and 2.16–3.91% recurrence) [19, 20]. However, microsurgical removal is thought to have a higher morbidity than endoscopic removal, including the development of infections, shunt necessity, and infarction [20–23]. The approach selected for each patient depends upon several factors including a surgeon’s preference and size of the colloid cyst, for instance neurosurgery may be recommended in symptomatic patients or those with cysts larger than 7 mm [24]. The non-invasive endoscopic approach was first offered as an alternative to microsurgery in 1982 and over the past 20 years has been gaining favor due to the believed increase in patient comfort and decreased morbidity and rates of complications [1, 20, 25]. The patients in our study had a similar proportion of microsurgical craniotomy and endoscopic approaches, with a slightly higher proportion undergoing microsurgical craniotomy.

Case reports show variable recovery and resolution of neuropsychiatric symptoms following cyst removal with some patients experiencing recurrence of their initial delusions and cognitive deficits [26]. A social media survey similar to our study, found that surgical removal of the colloid cyst improved quality of life and decreased headache disability [13]. However, there are cases in which patients develop neuropsychiatric symptoms when surrounding brain structures are damaged during surgery [7]. Most research on post-operative complications focuses on the association between bilateral interruption of the fornix structures and resulting memory defects, particularly in recall rather than recognition [27, 28]. Of the few studies conducting a neuropsychological assessment of colloid cyst patients following endoscopic removal, one study showed improvement in memory and quality of life (QOL) within 3–6 months after surgery compared to pre-surgical baseline [29]. Our study adds to this knowledge base by casting a wider net to a cross-section of colloid cyst patients regardless of time since surgery and identified persisting deficits. Our results suggest the greatest self-reported symptoms following any colloid cyst removal surgery were memory deficits affecting ~ 30%, followed by anxiety affecting ~ 20%. Worryingly, ~ 16% qualified for follow up regarding suicidal ideation and ~ 15% drank at least 4 drinks a day raising questions about undertreated and underrecognized mental health disorders and substance use. While there is no standardized protocol for treating and following-up colloid cyst patients, this suggests there is room to incorporate cognitive and psychiatric therapies. While a colloid cyst may be resolved, our findings suggested a high psychiatric morbidity that is underacknowledged.

Psychiatric conditions occur in high rates in those with central nervous system tumors and experienced by up to an estimated 50% of primary brain tumor patients. Furthermore, 93% of high-grade glioma patients demonstrate depression-like symptoms, with only 22% being identified as having depression by physicians [30, 31]. Patients with rare disease have a higher incidence of mental health disorders, and often have their psychiatric symptoms misdiagnosed. Among rare disease patients, a reported 69% experienced depression, while 82% experienced stress and anxiety [32, 33]. Notably, while neurology or neurosurgery follow-up was high in our sample (> 80%), psychiatric follow-up was low (~ 10%). Given these findings, we propose the medical community adopt a more long-term approach to treating colloid cyst patients and examine this phenomenon in similar patient populations extending to brain tumor and rare disease. Furthermore, the implementation of psychiatric screening tools as standard of care may identify those at risk for mental health conditions.

Beyond the physiological impact of CC and subsequent surgery, many other factors may leave a lasting impact on patients. A study of neuropsychiatric outcomes following surgical resection of colloid cysts shows that QOL is negatively impacted by perception of the neurosurgeon and their choices. Patients who felt a lack of confidence in their neurosurgeon, felt their concerns were dismissed by their neurosurgeon, or did not have the neurosurgeon recommend follow-up reported lower QOL [13]. Insufficient support by physicians is a common complaint amongst rare disease patients as they do not receive specific guidance for daily life, have subpar information regarding symptoms, and continue to experience lasting sequelae without support. For instance, qualitative interviews with Ehlers-Danlos syndrome patients experiencing chronic pain revealed major complaints regarding providers’ lack of support and knowledge and 53% reported using drugs like cannabis or opioids, while 93% sought nonmedical relief through the form of physical therapy, massage, and meditation [34]. This often leads rare disease patients to seek peer-patients for support and health information [35]. While the present study does not measure quality of life, the results show inadequate follow-up particularly for mental health in the setting of persisting symptoms with attempts to self-manage or self-medicate.

Conducting a survey on a social-media platform, allowed our study to reach colloid cyst patients on a national scale and potentially eliminate barriers and biases associated with site-based data collection. To our knowledge, this is the largest colloid cyst survivor survey to date. However, it is also important to note that conclusions drawn from our study may not be representative of all colloid cyst patients as it may have culled patients with particularly distressing or symptomatic experiences, may have been subject to survivorship bias since data from non-surviving patients could not be included, may have been subject to older age exclusion bias in terms of computer usage and abilities, and had socioeconomic implications, i.e., internet access was required. Additionally, our participant population overly represented White women. Without a centralized database, it is unclear if this is an effect of the study design or may be a true reflection of the demographics most impacted by colloid cyst. Notably, our survey of adult patients excludes the pediatric population who may have a more aggressive presentation, and among whom earlier detection may improve clinical outcomes [36]. Additionally, use of the validated DSM-5 screener gives an appropriate screenshot of each patient’s psychiatric symptoms over the two weeks prior to their participation in our survey to assess persisting sequalae but may not paint a comprehensive picture of the time or fluctuations surrounding their clinical diagnosis. It is important to note the optional nature of the DSM-5 screener in our survey as only 130 of 225 possible respondents completed this. An explanation may be respondent fatigue as well as self-selection by respondents receptive to discussing sensitive topics including mental health and substance use.

It is possible that the act of joining a support group as a potential coping mechanism may reflect participants’ neuropsychiatric wellbeing, positive or negative. A study of illness perception in patients with rare diseases (systemic lupus erythematosus, scleroderma, and myasthenia gravis) showed that patients who sought out health information related to their illness experienced more concern about their condition. Conversely, those who used patient organizations for information had a better understanding of their illness, and those who knew someone else with the same diagnosis experienced more positive illness perception [35]. Support groups have several reported benefits including but not limited to providing emotional support, providing a platform for open discussion, and teaching coping skills [37].

Future research will further characterize patients at high risk for deterioration as well as establish management protocols. While current colloid cyst management is limited to stereotactic aspiration, removal via craniotomy or endoscopy, ventriculoperitoneal shunting for hydrocephalus, and conservative management, new treatments may be on the horizon. A clinical trial has examined the efficacy of radioisotope 90yttrium colloid injections into the cyst to prevent further growth or reduce size [4, 38]. In addition, future efforts should include the creation of patient registries to monitor outcomes, tissue biobanks, and multicenter consortia to achieve the necessary sample size to address this rare and understudied diagnosis.

## Conclusions

To our knowledge this is the largest cross-sectional study querying clinical characteristics among colloid cyst survivors. Higher rates of post-surgical complications were observed in the hydrocephalus and craniotomy groups. Amongst all CC survivors, persisting symptoms were reported to be high including those concerning for memory, anxiety, depression, suicidality, and substance use. People experiencing brain surgery, even for benign tumors, need to be assessed for neuropsychiatric morbidity.

## Data Availability

The study dataset is available via emailing a request to the Corresponding author (Gustafson).

## Abbreviations

CC: Colloid cyst
CCSS: Colloid Cyst Symptoms Survey
CSF: Cerebrospinal fluid
QOL: Quality of life
